# Supplementary material for: What does the American public know about child marriage?
Source: PLoS One. 2020 Sep 23;15(9):e0238346. doi: 10.1371/journal.pone.0238346 (PMC7510990; doi:10.1371/journal.pone.0238346)
Supplement: S1 File — (PDF) [file pone.0238346.s002.pdf]

**Supplementary Table 1: Understandings of 'child marriage' by participant Sex (N=609)**				
	**Female**	**Male**	**Other**	**p***
N	359	247	3	
What is the legal threshold for child marriage - mean (s.d)	16.57 (2.02)	16.87 (1.95)	17.33 (1.15)	0.17
What age marks the end of childhood? - mean (s.d)	16.34 (4.82)	16.02 (2.81)	16.33 (4.04)	0.63
In which age range do most 'child marriage' occur? - n (%)				0.01
Under 10 years	27 (7.52)	9 (3.64)	0 (0.00)	
10-12 years	100 (27.86)	46 (18.62)	0 (0.00)	
13-15 years	149 (41.50)	116 (46.96)	1 (33.33)	
16-18 years	68 (18.94)	65 (26.32)	2 (66.67)	
19-21 years	15 (4.18)	11 (4.45)	0 (0.00)	
In how many US states is 'child marriage' legal? - mean (s.d)	6.38 (10.62)	7.93 (13.01)	12.33 (19.66)	0.20
In which region is 'child marriage' most common? - n (%)				0.81
Central Asia	21 (5.85)	16 (6.48)	0 (0.00)	
East Asia and Pacific	39 (10.86)	24 (9.72)	1 (33.33)	
Europe	4 (1.11)	4 (1.62)	0 (0.00)	
Middle East and North Africa	190 (52.92)	122 (49.39)	1 (33.33)	
North America	16 (4.46)	18 (7.29)	0 (0.00)	
South Asia	34 (9.47)	28 (11.34)	1 (33.33)	
Sub-Saharan Africa	55 (15.32)	35 (14.17)	0 (0.00)	
What proportion of women marry before age 18 in sub-Saharan Africa? - mean (s.d)	50.86 (22.68)	44.01 (22.04)	32.00 (17.09)	<0.001
What proportion of women marry before age 18 in South Asia? - mean (s.d)	43.56 (21.83)	37.81 (21.13)	33.33 (20.82)	0.01
What proportion of women marry before age 18 in the US? - mean (s.d)	16.87 (15.54)	13.04 (15.90)	17.33 (4.04)	0.01
What proportion of women married before age 18 in the US in the 1950s? - mean (s.d)	35.65 (20.41)	28.13 (20.27)	40.67 (25.72)	<0.001
How often are child marriage forced? - n (%)				0.01
Always	126 (35.10)	56 (22.67)	1 (33.33)	
Most of the time	214 (59.61)	162 (65.59)	2 (66.67)	
Rarely	19 (5.29)	27 (10.93)	0 (0.00)	
Never	0 (0.00)	2 (0.81)	0 (0.00)	
* p-value from chi-squared test for categorical variables and ANOVA for continuous variables				

**Supplementary Table 2: Understandings of 'child marriage' by participant age (N=609)**					
	**<30**	**30s**	**40s**	**50+**	**p***
N	196	202	100	111	
What is the legal threshold for child marriage - mean (s.d)	16.71 (2.18)	16.94 (1.69)	16.45 (2.21)	16.45 (1.94)	0.10
What age marks the end of childhood? - mean (s.d)	16.49 (6.00)	15.71 (2.72)	16.18 (3.13)	16.64 (2.66)	0.17
In which age range do most 'child marriage' occur? - n (%)					0.02
Under 10 years	11 (5.61)	6 (2.97)	6 (6.00)	13 (11.71)	
10-12 years	54 (27.55)	47 (23.27)	24 (24.00)	21 (18.92)	
13-15 years	78 (39.80)	92 (45.54)	49 (49.00)	47 (42.34)	
16-18 years	41 (20.92)	52 (25.74)	14 (14.00)	28 (25.23)	
19-21 years	12 (6.12)	5 (2.48)	7 (7.00)	2 (1.80)	
In how many US states is 'child marriage' legal? - mean (s.d)	6.34 (11.57)	6.75 (10.55)	9.74 (14.22)	6.34 (11.22)	0.09
In which region is 'child marriage' most common? - n (%)					0.15
Central Asia	11 (5.61)	10 (4.95)	10 (10.00)	6 (5.41)	
East Asia and Pacific	25 (12.76)	16 (7.92)	11 (11.00)	12 (10.81)	
Europe	4 (2.04)	3 (1.49)	1 (1.00)	0 (0.00)	
Middle East and North Africa	92 (46.94)	122 (60.40)	41 (41.00)	58 (52.25)	
North America	17 (8.67)	7 (3.47)	6 (6.00)	4 (3.60)	
South Asia	23 (11.73)	15 (7.43)	13 (13.00)	12 (10.81)	
Sub-Saharan Africa	24 (12.24)	29 (14.36)	18 (18.00)	19 (17.12)	
What proportion of women marry before age 18 in sub-Saharan Africa? - mean (s.d)	44.19 (21.15)	47.07 (22.85)	50.73 (23.53)	53.88 (22.82)	0.002
What proportion of women marry before age 18 in South Asia? - mean (s.d)	40.61 (20.19)	38.09 (22.04)	44.35 (22.72)	44.95 (22.07)	0.02
What proportion of women marry before age 18 in the US? - mean (s.d)	15.79 (16.58)	13.92 (14.46)	16.91 (18.06)	15.61 (14.20)	0.42
What proportion of women married before age 18 in the US in the 1950s? - mean (s.d)	35.44 (20.91)	32.64 (19.87)	32.05 (21.03)	28.08 (20.82)	0.03
How often are child marriage forced? - n (%)					0.17
Always	69 (35.20)	51 (25.25)	36 (36.00)	27 (24.32)	
Most of the time	110 (56.12)	138 (68.32)	58 (58.00)	72 (64.86)	
Rarely	17 (8.67)	12 (5.94)	6 (6.00)	11 (9.91)	
Never	0 (0.00)	1 (0.50)	0 (0.00)	1 (0.90)	
* p-value from chi-squared test for categorical variables and ANOVA for continuous variables					

Supplementary Table 3: Understandings of 'child marriage' by highest level of education (N=609)						
	**High school**	**Some college**	**Associate's degree**	**Bachelor's degree**	**Masters/ doctoral degree**	**p***
N	62	146	66	264	71	
What is the legal threshold for child marriage - mean (s.d)	17.02 (1.87)	16.86 (1.50)	16.58 (1.99)	16.67 (2.12)	16.27 (2.47)	0.19
What age marks the end of childhood? - mean (s.d)	17.65 (9.70)	16.27 (3.02)	16.44 (2.61)	15.77 (2.86)	16.27 (2.66)	0.03
In which age range do most 'child marriage' occur? - n (%)						0.37
Under 10 years	4 (6.45)	11 (7.53)	7 (10.61)	12 (4.55)	2 (2.82)	
10-12 years	12 (19.35)	41 (28.08)	15 (22.73)	56 (21.21)	22 (30.99)	
13-15 years	25 (40.32)	57 (39.04)	26 (39.39)	127 (48.11)	31 (43.66)	
16-18 years	18 (29.03)	33 (22.60)	17 (25.76)	54 (20.45)	13 (18.31)	
19-21 years	3 (4.84)	4 (2.74)	1 (1.52)	15 (5.68)	3 (4.23)	
In how many US states is 'child marriage' legal? - mean (s.d)	3.29 (5.05)	5.65 (11.07)	5.91 (11.07)	8.14 (12.24)	10.08 (14.29)	0.003
In which region is 'child marriage' most common? - n (%)						0.03
Central Asia	4 (6.45)	6 (4.11)	9 (13.64)	12 (4.55)	6 (8.45)	
East Asia and Pacific	5 (8.06)	16 (10.96)	4 (6.06)	26 (9.85)	13 (18.31)	
Europe	0 (0.00)	1 (0.68)	0 (0.00)	4 (1.52)	3 (4.23)	
Middle East and North Africa	42 (67.74)	81 (55.48)	30 (45.45)	130 (49.24)	30 (42.25)	
North America	5 (8.06)	8 (5.48)	2 (3.03)	15 (5.68)	4 (5.63)	
South Asia	4 (6.45)	13 (8.90)	9 (13.64)	32 (12.12)	5 (7.04)	
Sub-Saharan Africa	2 (3.23)	21 (14.38)	12 (18.18)	45 (17.05)	10 (14.08)	
What proportion of women marry before age 18 in sub-Saharan Africa? - mean (s.d)	50.02 (25.64)	50.08 (22.28)	46.14 (24.43)	47.07 (21.85)	47.06 (22.06)	0.60
What proportion of women marry before age 18 in South Asia? - mean (s.d)	44.06 (23.12)	42.07 (22.05)	40.59 (21.91)	39.90 (20.30)	42.13 (24.63)	0.66
What proportion of women marry before age 18 in the US? - mean (s.d)	18.48 (17.92)	13.98 (11.59)	17.21 (16.74)	14.93 (16.23)	15.00 (18.18)	0.32
What proportion of women married before age 18 in the US in the 1950s? - mean (s.d)	33.27 (21.50)	32.82 (20.31)	32.59 (22.70)	32.91 (20.64)	30.59 (19.29)	0.94
How often are child marriage forced? - n (%)						0.33
Always	18 (29.03)	50 (34.25)	17 (25.76)	78 (29.55)	20 (28.17)	
Most of the time	36 (58.06)	91 (62.33)	45 (68.18)	160 (60.61)	46 (64.79)	
Rarely	8 (12.90)	5 (3.42)	3 (4.55)	25 (9.47)	5 (7.04)	
Never	0 (0.00)	0 (0.00)	1 (1.52)	1 (0.38)	0 (0.00)	

* p-value from chi-squared test for categorical variables and ANOVA for continuous variables

**Supplementary Table 4: Understandings of 'child marriage' by employment status (N=609)**					
	**Unemployed**	**Employed**	**Student**	**Retired**	**P***
N	76	479	32	22	
What is the legal threshold for child marriage - mean (s.d)	16.84 (1.35)	16.72 (2.08)	16.72 (1.11)	15.68 (2.66)	0.11
What age marks the end of childhood? - mean (s.d)	15.83 (3.12)	16.16 (4.33)	17.50 (3.41)	16.64 (3.02)	0.25
In which age range do most 'child marriage' occur? - n (%)					0.13
Under 10 years	4 (5.26)	27 (5.64)	2 (6.25)	3 (13.64)	
10-12 years	27 (35.53)	103 (21.50)	11 (34.38)	5 (22.73)	
13-15 years	29 (38.16)	216 (45.09)	10 (31.25)	11 (50.00)	
16-18 years	15 (19.74)	108 (22.55)	9 (28.12)	3 (13.64)	
19-21 years	1 (1.32)	25 (5.22)	0 (0.00)	0 (0.00)	
In how many US states is 'child marriage' legal? - mean (s.d)	6.37 (10.07)	7.20 (11.84)	5.88 (11.83)	7.41 (14.18)	0.88
In which region is 'child marriage' most common? - n (%)					0.27
Central Asia	2 (2.63)	34 (7.10)	0 (0.00)	1 (4.55)	
East Asia and Pacific	9 (11.84)	51 (10.65)	3 (9.38)	1 (4.55)	
Europe	0 (0.00)	7 (1.46)	1 (3.12)	0 (0.00)	
Middle East and North Africa	51 (67.11)	236 (49.27)	16 (50.00)	10 (45.45)	
North America	2 (2.63)	30 (6.26)	2 (6.25)	0 (0.00)	
South Asia	5 (6.58)	50 (10.44)	4 (12.50)	4 (18.18)	
Sub-Saharan Africa	7 (9.21)	71 (14.82)	6 (18.75)	6 (27.27)	
What proportion of women marry before age 18 in sub-Saharan Africa? - mean (s.d)	51.92 (23.66)	46.48 (22.39)	45.91 (18.98)	70.27 (16.66)	<0.001
What proportion of women marry before age 18 in South Asia? - mean (s.d)	42.88 (22.52)	40.16 (21.34)	36.78 (20.14)	63.91 (15.94)	<0.001
What proportion of women marry before age 18 in the US? - mean (s.d)	12.67 (9.90)	15.82 (16.97)	12.22 (9.59)	18.05 (9.28)	0.21
What proportion of women married before age 18 in the US in the 1950s? - mean (s.d)	31.72 (18.53)	32.63 (21.19)	35.81 (20.26)	31.00 (17.57)	0.79
How often are child marriage forced? - n (%)					0.81
Always	24 (31.58)	145 (30.27)	11 (34.38)	3 (13.64)	
Most of the time	48 (63.16)	293 (61.17)	20 (62.50)	17 (77.27)	
Rarely	4 (5.26)	39 (8.14)	1 (3.12)	2 (9.09)	
Never	0 (0.00)	2 (0.42)	0 (0.00)	0 (0.00)	
* p-value from chi-squared test for categorical variables and ANOVA for continuous variables					

Supplementary Table 5: Understandings of 'child marriage' by political leanings (N=609)						
	**Very liberal (0-20)**	**Liberal (21-40)**	**Moderate (41-60)**	**Conservative (61-80)**	**Very conservative (81-100)**	**P***
N	202	77	170	63	97	
What is the legal threshold for child marriage - mean (s.d)	16.67 (1.69)	16.69 (1.54)	16.88 (1.74)	16.08 (2.97)	16.84 (2.45)	0.10
What age marks the end of childhood? - mean (s.d)	16.00 (2.71)	16.68 (2.64)	16.62 (6.29)	15.65 (2.98)	15.91 (3.22)	0.30
In which age range do most 'child marriage' occur? - n (%)						0.003
Under 10 years	5 (2.48)	3 (3.90)	13 (7.65)	7 (11.11)	8 (8.25)	
10-12 years	49 (24.26)	23 (29.87)	39 (22.94)	12 (19.05)	23 (23.71)	
13-15 years	109 (53.96)	32 (41.56)	66 (38.82)	28 (44.44)	31 (31.96)	
16-18 years	34 (16.83)	17 (22.08)	46 (27.06)	13 (20.63)	25 (25.77)	
19-21 years	5 (2.48)	2 (2.60)	6 (3.53)	3 (4.76)	10 (10.31)	
In how many US states is 'child marriage' legal? - mean (s.d)	7.19 (11.84)	8.43 (13.16)	5.62 (10.34)	7.70 (12.58)	7.66 (11.83)	0.39
In which region is 'child marriage' most common? - n (%)						0.91
Central Asia	12 (5.94)	4 (5.19)	11 (6.47)	5 (7.94)	5 (5.15)	
East Asia and Pacific	22 (10.89)	10 (12.99)	20 (11.76)	3 (4.76)	9 (9.28)	
Europe	2 (0.99)	1 (1.30)	1 (0.59)	2 (3.17)	2 (2.06)	
Middle East and North Africa	99 (49.01)	44 (57.14)	92 (54.12)	29 (46.03)	49 (50.52)	
North America	11 (5.45)	2 (2.60)	7 (4.12)	6 (9.52)	8 (8.25)	
South Asia	24 (11.88)	8 (10.39)	16 (9.41)	6 (9.52)	9 (9.28)	
Sub-Saharan Africa	32 (15.84)	8 (10.39)	23 (13.53)	12 (19.05)	15 (15.46)	
What proportion of women marry before age 18 in sub-Saharan Africa? - mean (s.d)	46.05 (21.90)	51.71 (22.58)	45.09 (23.34)	51.65 (21.74)	51.76 (22.80)	0.92
What proportion of women marry before age 18 in South Asia? - mean (s.d)	38.58 (20.58)	43.35 (22.74)	38.01 (20.68)	48.54 (22.85)	45.64 (22.48)	<0.001
What proportion of women marry before age 18 in the US? - mean (s.d)	13.47 (13.93)	14.74 (14.15)	14.05 (14.31)	21.35 (20.01)	17.95 (18.61)	0.003
What proportion of women married before age 18 in the US in the 1950s? - mean (s.d)	31.33 (19.03)	32.00 (20.14)	31.61 (21.21)	38.48 (21.48)	33.76 (22.58)	0.16
How often are child marriage forced? - n (%)						0.16
Always	68 (33.66)	26 (33.77)	54 (31.76)	10 (15.87)	25 (25.77)	
Most of the time	117 (57.92)	47 (61.04)	106 (62.35)	47 (74.60)	61 (62.89)	
Rarely	17 (8.42)	4 (5.19)	10 (5.88)	5 (7.94)	10 (10.31)	
Never	0 (0.00)	0 (0.00)	0 (0.00)	1 (1.59)	1 (1.03)	
* p-value from chi-squared test for categorical variables and ANOVA for continuous variables						
